# From Cooperation to Governance: Institutionalizing Health Cooperation under the China–Pakistan Economic Corridor

**DOI:** 10.12669/pjms.42.6.19598

**Published:** 2026-06

**Authors:** Zhang Tong, Qu Qiumei

**Affiliations:** 1 Zhang Tong, Lecturer, College of Humanities, Heilongjiang Dongfang University, Harbin, China; 2 Qu Qiumei, Visiting Lecturer, Department of History & Pakistan Studies, University of the Punjab, Lahore, Pakistan

**Keywords:** China-Pakistan Economic Corridor, institutionalized governance, operational mechanisms, project collaboration, sustainability

## Abstract

This study moves beyond examining individual projects and infrastructure outcomes to explore how health cooperation under the China-Pakistan Economic Corridor has evolved into a stable and institutionalized governance mechanism. It addresses key questions: How has health cooperation progressed from project-based collaboration to institutionalized governance? What mechanisms support this institutionalization? How does such cooperation contribute to sustainable health governance structures?

The study synthesizes evidence on cooperative practices, operational mechanisms, and sustainability outcomes to explain how health cooperation under CPEC has gradually developed into a long-term governance framework. By proposing a “Model of Health Governance Institutionalization,” it demonstrates how bilateral health cooperation can evolve into sustainable governance structures in developing countries, contributing to research in global health governance.

## INTRODUCTION

Research shows that wealthier countries generally achieve better health outcomes because they invest more in healthcare and preventive services. In this context, the economic gains generated through the China-Pakistan Economic Corridor (CPEC) can also lead to significant improvements in public health. The proposed China-Pakistan Health Corridor (CPHC) could therefore become an important component of CPEC.

China and Pakistan have already achieved notable success under CPEC in infrastructure, energy, and green development. Expanding this cooperation to the health sector would strengthen public health systems, improve healthcare access, and support economic and social development in both countries. As trade and people-to-people exchanges increase, the risk of cross-border infectious diseases also rises, making stronger bilateral cooperation in public health and emergency preparedness essential.

Healthcare remains a major priority for both governments and is widely accepted as an important area of collaboration under CPEC. Health cooperation not only improves the well-being of the people but also strengthens policy coordination, infrastructure connectivity, trade, and mutual understanding between the two countries. Academic exchanges, medical training, and joint healthcare projects can further deepen this partnership while creating broader public support for CPEC.

China’s healthcare sector has made remarkable progress over the past decades and offers valuable lessons for developing countries. Traditional Chinese Medicine (TCM), which historically formed part of the ancient Silk Road, has gained increasing acceptance in Pakistan. Promoting health diplomacy, medical exchanges, and TCM cooperation can further strengthen China’s role in regional and global health governance.

At the same time, expanding China-Pakistan health cooperation creates opportunities for collaboration in pharmaceuticals, medical devices, telemedicine, health informatics, medical tourism, and biotechnology. Such cooperation can improve healthcare delivery, encourage innovation, boost trade in health services, and contribute to economic growth in both countries.

## METHODOLOGY

This study examines the institutionalization of health cooperation under the China-Pakistan Economic Corridor. Using qualitative literature analysis and comparative policy evaluation, it proposes a model explaining how bilateral health cooperation can evolve into sustainable governance structures in developing countries.

The study is based on official policy documents issued by Chinese government institutions, including the State Council, National Development and Reform Commission, Ministry of Foreign Affairs, and National Health Commission. It also utilizes reports from the World Health Organization (WHO), Pakistan’s Ministry of Planning, the Chinese Embassy in Pakistan, and the National Agency for International Development Cooperation. Additional information was obtained from academic institutions, policy organizations, and media sources including China Daily, Xinhua News Agency, Today Pakistan, and China Economic Net.

Sources were selected on the basis of credibility, relevance, and their contribution to understanding the evolution of China-Pakistan health cooperation from project-based collaboration to long-term institutional governance.

### Evolution of Health Cooperation under CPEC^1^:

Health cooperation between China and Pakistan has expanded steadily over the past decade. In 2016, the Pakistan Society of Interventional Cardiology joined a Chinese training programme that enabled Pakistani doctors to benefit from advanced cardiac intervention technologies and expertise.

In May 2017, the China-Pakistan Benevolence Medical Emergency Center was inaugurated at Gwadar Port with support from the Red Cross Society of China. The center provides emergency healthcare services to local residents and Chinese workers in the region. In 2018, the “Health Express: Bringing Light to Pakistan” initiative provided eye treatment to five hundred Pakistani patients while also offering surgical training opportunities to Pakistani ophthalmologists.

During the COVID-19 pandemic in 2020, Chinese medical experts visited Pakistan to share experience in epidemic prevention and treatment protocols. In the same year, both countries signed a memorandum of understanding on high-end medical cooperation focusing on artificial intelligence, medical imaging, training, and nationwide healthcare services.

Cooperation in traditional medicine has also expanded. Pakistani representatives at the 2021 Shanghai Cooperation Organization Forum on Traditional Medicine expressed interest in joint clinical research and collaboration in TCM. China and Pakistan also cooperated in vaccine development and production, enabling local vaccine filling in Pakistan.

In March 2023, the China-Pakistan Joint AI Medical Diagnostic Laboratory was launched at Akbar Niazi Teaching Hospital in Islamabad to provide free cervical cancer screening for Pakistani women. Chinese medical technology companies supported the project through equipment and consumables.

Traditional Chinese Medicine and acupuncture are becoming increasingly popular in Pakistan, promoting medical exchange and providing patients with additional treatment options. In 2023, both countries also discussed cooperation in TCM education, research, and professional training.

In December 2023, the China-Pakistan Friendship Hospital in Gwadar, a major CPEC livelihood project funded by China, was completed. In January 2024, the China-Pakistan Medical and Surgical Equipment B2B Conference in Beijing encouraged greater investment and technical cooperation in Pakistan’s medical device sector through incentives such as tax exemptions and simplified approval procedures.^2^,^3^

### Surgery for Congenital Heart Diseases:

Further cooperation agreements were signed in 2024 and 2025 covering medical research, surgical services, telemedicine, artificial intelligence, infectious disease management, hospital administration, and healthcare training. Chinese medical teams successfully performed congenital heart surgeries for Pakistani children in early 2025, marking the first bilateral technical cooperation in this field.^4^

Health experts have emphasized that healthcare facilities along the CPEC route can improve regional health security, support tourism, and strengthen pharmaceutical cooperation. Collaboration in bioengineering, data analytics, and health information systems could also help address major challenges facing Pakistan’s healthcare sector.

In 2025, cooperation further expanded through telemedicine initiatives, strategic hospital partnerships, TCM training programmes for Pakistani doctors in China, and the introduction of Chinese biosimilar medicines into Pakistan’s healthcare market. Hospitals in Kashgar and Gilgit also launched telemedicine and infectious disease cooperation programmes.

### China-Pakistan International Neurosurgery Forum:

In January 2026, the first China-Pakistan International Neurosurgery Forum was held at Wuhan University. Pakistan also adopted Alibaba DAMO Academy’s AI-based multi-cancer screening technology, enabling earlier detection of major cancers and chronic diseases.

Overall, China-Pakistan health cooperation has evolved from emergency medical assistance and small-scale welfare projects to broader institutional collaboration involving healthcare infrastructure, medical technology, training, artificial intelligence, public health, and pharmaceutical cooperation. These initiatives continue to improve healthcare access and strengthen bilateral relations under the Belt and Road Initiative.

### People’s Livelihood Corridor:

The “Action Plan for Building a Closer China-Pakistan Community with a Shared Future in the New Era (2025–2029)” further institutionalizes health cooperation under CPEC’s “People’s Livelihood Corridor.” The plan emphasizes telemedicine, infectious disease surveillance, vaccine cooperation, traditional medicine, AI-assisted diagnosis, medical imaging platforms, and joint public health emergency preparedness.^3^

The framework also promotes mutual recognition of drug registration and medical device standards, training programmes for Pakistani healthcare professionals in China, deployment of Chinese medical experts to Pakistan, and remote continuing medical education. This shift from project-based aid to long-term institutional and mutually beneficial cooperation may provide an important model for regional and global health governance under the Belt and Road Initiative.

### Mechanisms of Institutionalization:

Under the China-Pakistan Economic Corridor (CPEC), health cooperation should combine government coordination with the active participation of public institutions, enterprises, and civil society organizations. Collaboration with Pakistani NGOs, community organizations, and grassroots groups can strengthen academic exchanges, medical services, and charitable healthcare initiatives. Through joint efforts of governments and civil society, both countries can promote the health and well-being of their populations.

China and Pakistan have expanded cooperation through government exchanges, institutional partnerships, and personnel training. China has provided medical assistance through long- and short-term medical teams, emergency rescue support during disasters, and initiatives such as the “Brightness Mission” for free cataract surgeries. These programs combine humanitarian assistance with long-term healthcare development.

### Establishment of Cooperation Mechanisms:

Both countries should strengthen high-level health exchanges and formalize cooperation through agreements and institutional forums under the CPEC framework. Proposed initiatives include the “Silk Road Health Cooperation Forum,” the “China-Pakistan Youth Unity Circle,” and the “International Medical Innovation Cooperation Forum.” Chinese and Pakistani medical institutions are also encouraged to sign memorandums of understanding to enhance collaboration.

China’s overseas medical missions and volunteer programs have earned significant recognition in Pakistan. In May 2023, Chinese and Pakistani volunteers established a “Home of Love” in Islamabad to support poor families seeking treatment for sick children by providing free accommodation, meals, and medical assistance.

At the 2025 International Forum on Medical Innovation and Cooperation, Pakistan’s Federal Health Minister, Mustafa Kamal, invited Chinese pharmaceutical and medical device companies to establish manufacturing facilities in Pakistan, assuring them of government support and investment opportunities.

### Infectious Disease Prevention and Control:

China and Pakistan should establish a joint information-sharing and disease surveillance mechanism for common and emerging infectious diseases. Priority areas include HIV/AIDS, malaria, dengue, tuberculosis, avian influenza, and polio eradication, along with zoonotic diseases such as echinococcosis and plague. Both countries should strengthen outbreak reporting systems, emergency coordination mechanisms, and rapid response capabilities.

During the COVID-19 pandemic, Pakistan provided China with emergency medical supplies in February 2020. Later, when Pakistan faced a severe outbreak, China responded with medical teams, equipment, testing kits, ventilators, and financial and technical support. Chinese public and private organizations, including Alibaba, Haier, and several state-owned enterprises, also donated large quantities of medical supplies.

Since 2016, the Wuhan Institute of Virology and the Bioresearch Center in Karachi have collaborated on research into emerging infectious diseases along the CPEC route. Their work focuses on disease surveillance, genetic evolution of viruses, and strengthening biosecurity and rapid response systems.^5^,^6^

### Capacity Building and Talent Development:

China and Pakistan should enhance cooperation in medical education, public health training, and professional exchanges. Training bases can be established in several Chinese provinces, while short- and long-term training programs can strengthen Pakistan’s healthcare workforce.

### China-Pakistan Medical University Alliance:

The China-Pakistan Action Plan (2025–2029) includes 3,000 training and study opportunities for Pakistani citizens in areas including medicine and public health. Plans also include developing a China-Pakistan Medical University Alliance and expanding cooperation among hospitals, universities, and research institutions.

China’s pediatric congenital heart disease treatment project in Pakistan has been widely appreciated and reflects China’s growing contribution to global healthcare and medical innovation.

**Figure:**
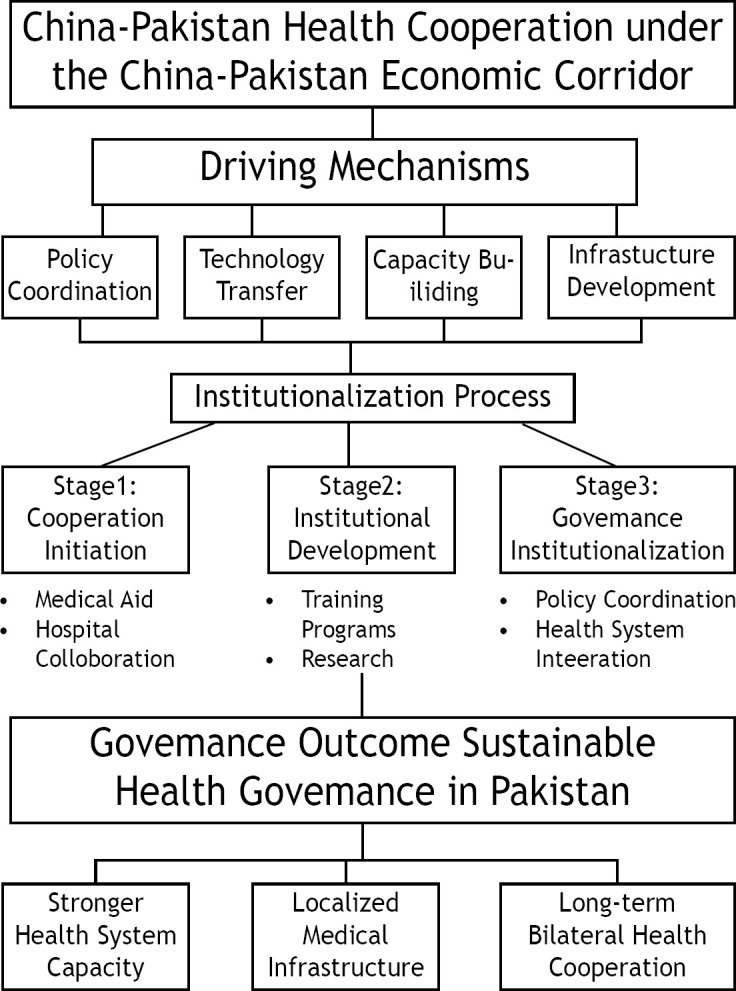


### Public Health Emergency Response and Medical Assistance:

China and Pakistan should expand cooperation in public health emergency preparedness through joint drills, coordinated emergency response mechanisms, and rapid medical assistance programs. China can continue dispatching short-term medical teams and supplying emergency medicines and protective equipment when required.

In December 2024, the Chinese Consulate General in Karachi donated medical supplies to the Gwadar China-Pakistan Friendship Hospital, a flagship CPEC healthcare project equipped with modern laboratories, CT scanners, and ultrasound machines donated by the Chinese government.

Chinese medical teams have also served in Gwadar since 2017, treating local residents and Chinese workers while conducting health education and training programs for port employees and students.

### Traditional Medicine Cooperation:

China and Pakistan have expanded cooperation in traditional medicine, particularly Traditional Chinese Medicine (TCM) and herbal medicine. Cooperation includes healthcare services, education, scientific research, industrial collaboration, and standard-setting initiatives.

The 2025 China-Pakistan Traditional Medicine Online Exchange Conference promoted institutional cooperation and opened new channels for trade and research collaboration. In 2020, the China-Pakistan Traditional Chinese Medicine Cooperation Center was established in Huaihua to support research, product registration, clinical studies, professional training, and bilateral exchanges in traditional medicine.

### Healthcare Systems and Policy Cooperation:

Both countries should establish long-term cooperation on healthcare systems, universal health coverage, health legislation, aging populations, and health policy reforms. China’s experience in healthcare reform and policy development can provide valuable lessons for Pakistan through academic exchanges and collaborative policy research.

### Health Development Assistance:

China continues to support Pakistan through medical teams, healthcare experts, infrastructure development, donations of medicines and equipment, training programs, and outreach initiatives such as the “Brightness Mission.” These activities strengthen Pakistan’s healthcare delivery system while deepening bilateral cooperation.

### Development of the Health Industry:

China and Pakistan should promote cooperation in medical tourism, telemedicine, pharmaceuticals, and medical devices. Establishing cross-border telemedicine networks and encouraging Chinese pharmaceutical companies to invest in Pakistan can improve access to healthcare services and high-quality medical products. Reducing trade barriers and encouraging bilateral investment can further support the growth of the health industry.

## CONCLUSIONS

Health cooperation under CPEC has evolved from project-based medical assistance into a more comprehensive and institutionalized governance framework. This transformation can be understood in three phases:

***Medical Cooperation*** – medical aid, hospital partnerships, and clinical services.

***Institutional Development*** – training programs, telemedicine, and research collaboration.

***Governance Integration*** – policy coordination, health system reform, and institutional cooperation.

The institutionalization of China-Pakistan health cooperation is reflected in the development of joint mechanisms for infectious disease control, emergency response, professional training, and policy coordination. Capacity-building initiatives, knowledge transfer, and professional exchanges have strengthened Pakistan’s healthcare system and improved resilience.

### This study identifies eight major areas for future cooperation:

- institutional mechanisms,
- infectious disease prevention and control,
- capacity building and talent development,
- emergency medical response,
- traditional medicine,
- healthcare systems and policies,
- health development assistance, and
- health industry cooperation.

Traditional medicine cooperation has expanded the cultural and scientific dimensions of bilateral collaboration, while increasing policy coordination reflects a shift from technical cooperation toward deeper governance integration.

Overall, health cooperation under CPEC has moved beyond isolated projects toward a stable and sustainable institutional framework. Future cooperation should prioritize sustainability, policy coordination, local capacity-building, and effective evaluation mechanisms to ensure equitable and long-term healthcare development for both countries.
